# The Impact of Living in Housing With Care and Support on Loneliness and Social Isolation: Findings From a Resident-Based Survey

**DOI:** 10.1093/geroni/igac061

**Published:** 2022-09-29

**Authors:** Brian Beach, Paul Willis, Jillian Powell, Alex Vickery, Randall Smith, Ailsa Cameron

**Affiliations:** Research Department of Epidemiology and Public Health, University College London, London, UK; School for Policy Studies, University of Bristol, Bristol, UK; School for Policy Studies, University of Bristol, Bristol, UK; School for Policy Studies, University of Bristol, Bristol, UK; School for Policy Studies, University of Bristol, Bristol, UK; School for Policy Studies, University of Bristol, Bristol, UK

**Keywords:** Housing with care, Inclusive communities, Loneliness, Propensity score matching, Social connections

## Abstract

**Background and Objectives:**

Housing with care is often lauded as a way to combat loneliness and social isolation in later life. This study examined whether housing with care created better outcomes for residents in terms of loneliness and social isolation than they might expect if they were living in the community.

**Research Design and Methods:**

A survey was distributed to residents of housing with care as part of the Diversity in Care Environments project. It was designed to enable comparison with the English Longitudinal Study of Ageing. Propensity score matching was applied to identify the effect of housing with care residence on loneliness and social isolation.

**Results:**

People living in housing with care had lower levels of loneliness than would be expected if they lived in the general community, with an average treatment effect on the treated (ATT) of −0.407 (95% CI = −0.601, −0.214). In contrast, social isolation was found to be slightly higher for residents than would be expected if they were in the community (ATT = 0.134 [95% CI = 0.022, 0.247]). Higher social isolation appears driven by less frequent contact with friends and reduced organizational membership rather than any difference in contact with family and children.

**Discussion and Implications:**

Our research has shown a positive impact on subjective social experiences from housing with care residence, despite a slight increase in objective social isolation. The findings underscore the importance of looking at loneliness and social isolation as distinct concepts as well as the effectiveness of housing with care at improving later-life outcomes.

**Translational Significance:** Housing with care schemes are promoted to reduce loneliness and social isolation, which, in turn, would alleviate other pressures linked to health and care outcomes. Using data from a resident-based survey and a large nationally representative aging study, we provide evidence that residents of housing with care have lower levels of loneliness than they would if they were living in nonspecialist housing. This evidence strengthens the case for housing with care, which should stimulate investment in and policymaking for the sector, generating more positive opportunities to support aging well for people in later life.

Globally, population aging is taking place alongside increasing diversification in family structures, patterns of relationships, and identities. These social transformations have stimulated numerous debates in research, policy, and practice around how best to adapt to an aging society that will be characterized not only by a greater number of older people but also a more diverse (older) society. Specialist retirement housing has been posited as one solution to enable people to move through later life with both independence and support for changing needs (Barac & Park, 2009). A key ambition of such housing is to provide social spaces for residents that support opportunities for social engagement and help counter the risks of isolation and loneliness. However, there remains a gap in the evidence base on the extent to which this ambition is successful, as no study has looked into housing with care schemes specifically using quantitative survey methods. To address this gap, this paper examines data collected from the research project, Diversity in Care Environments (DICE), drawing on a bespoke survey among housing with care residents designed to facilitate comparative analyses with existing data covering older people living in the community, using a quantitative matching technique to make a valid comparison. This allows us to explore the impact that housing with care can have to protect against social isolation and loneliness among older people in England.

## Background and Objectives

Global policy and health care have increasingly favored the “aging in place” model for older populations (World Health Organization [WHO], 2007). This model supports people as they age to remain living within their communities, receiving care and support when necessary while retaining a level of autonomy and independence. Aging in place supports the notion that remaining in a familiar environment positively affects well-being, relationships, and experiences in later life (Van Dijk et al., 2015).

One’s home is clearly at the center of considerations related to aging in place, particularly as part of the ambition for aging in place is to help prevent moves into residential care homes (i.e., the traditional concept of nursing or care homes). Alongside efforts to strengthen older people’s ability to remain in their own homes for longer, new forms of housing evolved over the 20th century that are tailored toward the needs of older people in a context of aging. In this research, we broadly refer to these models as housing with care.

### Housing in Later Life

In the United Kingdom, most older people live in general housing in mixed demographic communities (Ministry of Housing, Communities & Local Government, 2020). Many older people have strong attachments to their homes, wishing to remain there and to delay any transition into residential care or age-restricted housing, which can be perceived as a loss of independence (Bigonnesse & Chaudhury, 2021; Mulliner et al., 2020). Remaining at home has also been viewed as important for continuity of self-identity and social ties with friends, family, and neighbors (Stones & Gullifer, 2016).

However, as people age and become increasingly frail, the built environment can become a barrier to independence. Adaptations such as ramps and stairlifts can help people stay at home, but these are not always an adequate solution; structural limitations of some homes may not permit the installation of necessary equipment and technologies (Sixsmith & Sixsmith, 2008). Moreover, a fear of crime, declining health that impacts mobility, and poor local amenities (e.g., transportation) can create further barriers to independence, constraining older people to their home and reducing activity and social participation (Mulliner et al., 2020; Sixsmith & Sixsmith, 2008). For people experiencing age-related declines in health and mobility, the home environment can become a place of isolation and loneliness rather than of continued independence (Sixsmith & Sixsmith, 2008).

An evolving market of specialist housing for later life has emerged to provide a supportive environment for people to retain their autonomy and independence as they age. Housing with care includes models such as assisted living, extra-care housing, retirement homes, and sheltered housing (Care Quality Commission [CQC], 2015), but the terminology related to the sector varies widely both within and between countries, including housing for later life that does not explicitly incorporate care and support provision (Baker, 2002; Beach, 2018).

Despite ambiguities in the terms used for the broader sector of retirement living, models for housing with care generally share three key features: residents live in self-contained units, care is available on a permanent or temporary basis, and sites have communal facilities such as lounges and gardens (Evans et al., 2017). They differ from traditional care homes in that accommodation generally consists of purpose-built (or purpose-adapted) self-contained units that are either owned or rented under an occupancy agreement. Accommodation tends to be individual flats or bungalows, designed to facilitate the delivery of care to residents, either on entry or at some point in the future (CQC, 2015). Many schemes also feature organized social activities designed to promote social interactions among residents and the development of relationships and community (Blood & Pannell, 2012). Purposefully designed communal spaces facilitate the staging of these activities aimed at reducing social isolation and loneliness (Callaghan et al., 2009).

The promotion of housing with care aligns with broad UK policy aims related to housing and an aging society (Atkinson et al., 2014). One key aspect in this respect is how housing with care can promote independence and choice, enhancing quality of life for residents. Such policy ambitions align with the evolving emphasis on personalization in delivering care (Department of Health and Social Care, 2021), while housing with care offers an alternative to other residential settings that might contribute to social isolation (Callaghan & Towers, 2014). The potential for housing with care to combat loneliness and isolation in later life is often lauded as part of its broader social value.

### Social Isolation and Loneliness

Loneliness and social isolation have emerged as public health challenges for aging populations both within the United Kingdom (Bu et al., 2020) and internationally (Crowe et al., 2021). As people age and experience health deterioration, their risk of becoming socially isolated and experiencing loneliness increases (De Jong Gierveld et al., 2018). Moreover, social isolation can have a detrimental impact on both physical and mental health (Taylor, 2020), and loneliness has been linked to an increased risk of physical frailty (Gale et al., 2018). Lonely and socially isolated older people consistently experience a decline in mobility and cognitive function, verbal fluency, and the ability to carry out activities of daily living (Courtin & Knapp, 2017; Lara et al., 2019). Over time, the interaction between social isolation and loneliness can act to reinforce each other (Cacioppo & Cacioppo, 2018).

While related, loneliness and social isolation are two separate concepts. Factors associated with social isolation comprise both objective and subjective phenomena, such as living alone, having few social network ties, infrequent social contact, a lack of fulfilling social relationships, and/or a lack of sense of belonging (Cruise and Kee, 2017; Hand et al., 2014). Loneliness is more subjective, linked to an individual’s emotional perceptions of and responses to social isolation and lack of social connections (Fakoya et al., 2020; Griffiths, 2017). Social isolation is a risk factor in experiencing loneliness (Davidson and Rossall, 2015), but the concurrent experience of both is not inevitable. For example, older people who have support networks made up of close family, but few ties with friends and neighbors (i.e., family-dependent support networks) and those who have few ties with relatives, friends, and little community involvement (i.e., private restricted support networks) are thought to be more at risk of experiencing loneliness (Wenger, 1997; Wenger & Tucker, 2002).

Theoretical approaches to loneliness have included “attachment” perspectives (cf., Weiss, 1974), which postulate that different social relationships provide different emotional support (e.g., attachment, sense of worth). The “cognitive discrepancy” model of loneliness suggests that loneliness occurs where there is a discrepancy between individuals’ desired and achieved levels of social relationships (Perlman & Peplau, 1981). These discrepancies can especially be impacted by life events such as bereavement or onset of disability. Relationship expectations also feed into whether a person experiences loneliness, as a smaller social network made up of quality relationships may in fact be more rewarding emotionally than larger networks of poor quality or less desired social relationships (De Jong Gierveld et al., 2018).

Declining physical health is one of the most prominent factors in the loss of independence for older people, which can also compound social isolation (Abdi et al., 2019). A large meta-analysis of 70 independent prospective studies involving approximately 3.5 million participants concluded that actual and perceived social isolation can result in a higher likelihood of mortality (Holt-Lunstad et al., 2015). A more recent scoping review found that social isolation and loneliness consistently had a detrimental effect on physical and mental health in older age (Courtin & Knapp, 2017).

Direct effects have been found between satisfaction with social networks and loneliness, with feelings of loneliness and isolation directly related to attachment to neighborhoods (Kemperman et al., 2019). Attachment to neighborhoods and satisfaction with social networks were indirectly influenced by a feeling of safety within neighborhoods. Moreover, older adults who experience loneliness can often perceive their social environments as threatening and dangerous, which can lead to behaviors that negatively impact social interactions with family and friends (Cacioppo & Cacioppo, 2018).

To date, research examining the role of living environments in reducing loneliness and social isolation has largely focused on characteristics of neighborhoods (e.g., Kemperman et al., 2019) or comparisons of “aging in place” with traditional residential care homes (e.g., Stones & Gullifer, 2016). Other qualitative research has argued that extra-care and residential care settings enable social interdependencies to flourish, in contrast to loneliness and social isolation among people in the community (Hillcoat-Nallétamby, 2014). Other work has also highlighted the greater opportunity for extra-care settings to promote social interaction, but it did not find any difference in terms of loneliness across extra-care, residential, and community settings (Burholt et al., 2013). One study also highlighted the value of living in “a” community rather than “the” community as a means to avoid isolation (Croucher, 2008). Survey-based research in Sweden and Finland found that loneliness was higher among people aged 85+ living in institutional settings compared to those in the community (Nyqvist et al., 2013). The evidence for the impact of different residential settings on isolation and loneliness thus appears mixed, but studies are consistent in demonstrating the relationship between these outcomes and the living environment.

Such work follows an evolving theoretical tradition around how health and psychosocial well-being are influenced by one’s housing. Housing is recognized as a key social determinant of health within research and policy (Marmot et al., 2010; World Health Organization, 2018), and other work notes its link to subjective aspects of well-being (Clapham et al. 2018). Our work draws on this tradition, with consideration of more recent theories focused on social isolation and loneliness around meaningful interactions (Wigfield et al., 2022). The framework for meaningful interactions extends from symbolic interactionism to suggest that individuals’ experiences and circumstances (such as housing) impact their opportunities for social interactions that, in turn, influence their health and well-being.

To our knowledge, no study has quantitatively examined the impact that housing with care has in addressing loneliness and social isolation among residents when compared to those living in general community housing. The need to address this gap in the evidence base is further strengthened by a policy orientation that promotes such housing models as ways to enhance independence and social connections in later life. This research therefore addresses the following questions:

1. Are levels of loneliness lower among residents of housing with care compared to people living in general housing in the community?
2. Do residents of housing with care report lower levels of social isolation than similar people living in the community?

Based on the ethos of housing with care models, we hypothesize that loneliness and social isolation will both be lower for those living within housing with care settings.

## Research Design and Methods

### Data

The data used in this study were collected as part of the Diversity in Care Environments (DICE) study funded by the Economic and Social Research Council. DICE was a 3-year project exploring how housing with care schemes (i.e., specific housing with care communities) can support the social inclusion of older adults from different social groups and backgrounds. The project took a mixed-methods approach, employing a survey questionnaire of housing with care residents and qualitative interviews with residents from social minority backgrounds, housing staff, and key stakeholders. The study received ethical approval from the Faculty of Social Sciences and Law, University of Bristol.

The survey questionnaire was designed to collect a range of variables that would facilitate a matched analysis using the English Longitudinal Study of Ageing (ELSA; Banks et al., 2021). Such information, covering sociodemographic indicators (e.g., education, gender), health, social connections, and other psychosocial measures, was supplemented by questions specific to the experiences of people living in housing with care (e.g., on the living environment). Three providers of such schemes in England and Wales were project partners and assisted in the identification of schemes and the distribution of paper surveys to residents in all housing units across 104 schemes. Three thousand six hundred and ninety-four surveys were sent in late 2019 and early 2020, and completed surveys were returned directly to the research team using provided prepaid postage envelopes. Returned surveys arrived before the start of the COVID-19 lockdown in the United Kingdom in March 2020, resulting in 741 respondents from 94 schemes—a response rate of 22.4% of units when restricted to schemes where some surveys were returned.

### Methods

Our analysis focuses on two outcomes of interest: loneliness and social isolation, measured using established approaches. Loneliness reflects a composite score drawn from three questions (i.e., the UCLA three-item loneliness score; Hughes et al., 2004). Scores range from 3 to 9, where 9 is the highest level of loneliness; Cronbach’s α for our sample is .89 (and .83 for the ELSA sample). Social isolation used another composite score, combining items related to social networks and contacts. These items cover five areas: no partnership, no organizational membership, and less than monthly contact with children, other family members, or friends (Steptoe et al., 2013). Scores range from 0 to 5, with 5 reflecting the most isolation.

Our analysis aimed to compare the responses on our measures of interest among housing with care residents with the experiences of people living in the general community. To make a valid comparison, we drew on responses from Wave 9 of ELSA. We used propensity score matching (PSM) to create a comparison between the community-based sample from ELSA with our resident sample, reflecting key characteristics that impact the probability of residing in specialist housing. PSM can be used to estimate the impact of a “treatment” where randomization to a treatment or control group is not possible. Conceptually, PSM uses participant characteristics to match those receiving treatment and those not, enabling an estimate of the effects of the treatment that controls for the potential bias caused by differences between the two groups. The use of PSM for causal inference in observational studies is not without controversy (cf., King & Nielsen, 2019), though it remains popular in the social sciences (Rohrer, 2018). Moreover, PSM has a distinct advantage for bias correction over other quasi-experimental approaches, as it reduces the dimensions for matching to a single continuous covariate (Guo et al., 2020).

We built our models to estimate the propensity scores based on theoretical considerations rather than relying on any statistical goodness-of-fit measures (Black & Smith, 2004). In other words, we conduct our matching based on characteristics we reasonably believe shape whether people move into housing with care schemes in the United Kingdom. Our matching used measures for age, gender, ethnicity, self-reported health, the presence of chronic illness, whether living alone, and socioeconomic status; details are available in Supplementary Material.

Our approach considered data set membership as the treatment outcome, that is, residence in a housing with care scheme (our survey) was the treatment versus general community residence (ELSA) as control. Analyses were conducted using Stata 17.0 (StataCorp, 2021).

## Results

The prevalence of key characteristics among our sample is presented in Table 1. Although we have found no comprehensive data covering the full population of housing with care residents in England or Wales, our descriptive findings align with those of one large study of retirement housing residents (ProMatura International 2019). Table 1 also includes equivalent measures from ELSA, adjusted for survey design and restricted to those aged 55+ who completed the self-completion questionnaire (as questions on loneliness and social isolation were collected there) to illustrate comparability with our sample.

**Table 1.** Sociodemographic Characteristics of Study Samples

Sociodemographic characteristic	DICE	ELSA
	*N* (%)	%
Age		
Under 55	1 (0.14)	—
55–59	12 (1.62)	18.77
60–64	51 (6.90)	19.08
65–69	96 (12.99)	17.27
70–74	131 (17.73)	17.01
75–79	128 (17.32)	10.91
80–84	117 (15.83)	9.29
85–89	111 (15.02)	4.80
90+	92 (12.45)	2.87
Male	266 (36.49)	47.53
Female	463 (63.51)	52.47
Lives alone		
Yes	577 (78.72)	22.66
No	156 (21.28)	77.34
Ethnicity		
White	682 (96.06)	92.94
Mixed ethnic group	8 (1.13)	—
Black African	5 (0.70)	—
Black Caribbean	5 (0.70)	—
South Asian	3 (0.42)	—
East/South-East Asian	4 (0.56)	—
Other (not specified)	3 (0.42)	—
Activity-limiting chronic health problem		
Yes	465 (85.48)	32.54
No	79 (14.52)	67.46
Has children		
Yes	594 (83.54)	85.77
No	117 (16.46)	14.23
Has friends		
Yes	608 (88.37)	93.00
No	80 (11.63)	7.00
Housing tenure		
Own	160 (22.19)	81.34
Rent	543 (75.31)	18.50
Shared ownership	18 (2.50)	0.15

*Notes*: DICE = Diversity in Care Environments; ELSA = English Longitudinal Study of Ageing. ELSA figures are adjusted for survey design and are restricted to those aged 55+ and part of the self-completion ELSA sample. Some percentages do not total 100% due to rounding.

Table 2 provides an overview on the average scores among our sample and the unmatched ELSA sample across our key outcomes of interest: loneliness and social isolation. We have also included the component items for social isolation, as they reflect distinct aspects of social connections and may inform the way in which isolation varies. In these summary statistics, we see that our outcomes of interest look similar or worse among our survey respondents compared to the ELSA sample. However, comparing these pairs of outcomes does not account for the differences between residents of housing with care and those in the general community that likely shape experiences of loneliness and social isolation; this reiterates the importance of applying the matching technique.

**Table 2.** Summary Statistics on Outcomes of Interest

Outcome	DICE		ELSA (self-completion, 55+)	
	Mean	95% CI	Mean	95% CI
Loneliness score	4.34	4.20–4.48	4.15	4.10–4.20
Social isolation score	1.98	1.89–2.07	1.26	1.23–1.31
Components of social isolation score (%)				
No partner	79.0	76.0–81.9	30.7	29.2–32.1
Children (less than monthly contact)	23.8	20.6–26.9	24.5	23.1–25.9
Other family (less than monthly contact)	32.4	28.9–35.9	24.7	23.4–26.0
Friends (less than monthly contact)	22.7	19.5–25.8	15.2	14.0–16.3
No organizational membership	40.3	36.8–43.9	32.4	30.9–34.0

*Notes*: DICE = Diversity in Care Environments; ELSA = English Longitudinal Study of Ageing; CI = confidence interval. The ELSA analysis uses the Wave 9 self-completion sample aged 55+. Loneliness scores range 3–9, whereas social isolation ranges 0–5; higher scores relate to higher (i.e., worse) loneliness or isolation.

Complete results from our matching estimation are reported in Table 3. We report the average treatment effect on the treated (ATT) with 95% confidence intervals and *p*-values. The ATT provides an estimate of the average difference in our outcome of interest among those treated—those living in housing with care—compared with what would be expected if they were not treated (living in the general community). We also show two sets of results, with one excluding ethnicity as a covariate. Postestimation checks suggested that including ethnicity reduced the quality of the balance between our matching groups, assessed via standardized differences and variance ratios; details are provided in Supplementary Material. However, the results with and without ethnicity demonstrate the same trends.

**Table 3.** Results for Average Treatment Effect on the Treated (ATT)

Outcome	Including ethnicity			Excluding ethnicity		
	ATT	95% CI	*p*-value	ATT	95% CI	*p*-value
Loneliness score	−0.371	−0.569, −0.173	<.001	−0.407	−0.601, −0.214	<.001
Social isolation score	0.131	0.015, 0.246	.027	0.134	0.022, 0.247	.019
Components of social isolation score						
No partner	−0.010	−0.029, 0.011	.391	−0.009	−0.029, 0.011	.371
Children (less than monthly contact)	−0.025	−0.071, 0.021	.289	−0.019	−0.064, 0.026	.405
Other family (less than monthly contact)	0.021	−0.031, 0.074	.427	0.027	−0.026, 0.080	.318
Friends (less than monthly contact)	0.060	0.019, 0.102	.005	0.060	0.019, 0.101	.004
No organizational membership	0.073	0.020, 0.126	.007	0.067	0.012, 0.121	.016

We find strong evidence to support the hypothesis that people living in housing with care have lower levels of loneliness than would be expected if they lived in the general community. The ATT of −0.371 (95% CI −0.569, −0.173) indicates that the average score on the loneliness scale was over a third of a point lower. When conducting the matching estimate without ethnicity as a covariate, the reduction is even larger at −0.407 (95% CI −0.601, −0.214).

In contrast, social isolation was found to be higher for housing with care residents than would be expected if they were living in the community. The effect on the social isolation score was small but statistically significant, with an ATT of 0.131 (95% CI 0.015, 0.246) when including ethnicity and 0.134 (95% CI 0.022, 0.247) when excluding ethnicity. It is possible that these results indicate an isolating effect from living in such schemes; however, this estimated increase may relate less to schemes being more isolating overall and more about the changes in social networks and connections that occur for those who move into housing with care.

This is supported by looking at the separate components of the social isolation measure. We find significant treatment effects for contact with friends and for organizational membership, but not for the other measures related to the family, linking back to the concept of family-dependent social networks (e.g., Wenger, 1997). The ATT related to less than monthly contact with friends is 0.006 (95%CI 0.019, 0.102), while for no organizational membership it is 0.073 (95% CI 0.020, 0.126). Both results highlight a higher level of isolation on these measures than would be expected if they were living in the community.

The social isolation score includes a component related to whether the person has a partner. Given the high correlation between living alone and not having a partner, we also estimated effects for the social isolation score in two additional ways: excluding the covariate for living alone and using a modified social isolation score excluding the partnership component. We continued to exclude ethnicity, as the issues with balance persisted. Excluding “no partnership” from the measure of social isolation had a marginal effect, with the estimated ATT increasing to 0.161 (95% CI 0.051, 0.270). When removing living alone as a covariate, however, we see notable changes in the estimated ATT for social isolation and the partnership component (see Table 4).

**Table 4.** Results for Social Isolation Using Alternative Specifications Regarding Partnership and Living Alone

Outcome	ATT	95% CI	*p*-value
Social isolation score (excluding living alone covariate)	0.513	0.400, 0.626	<.001
Social isolation score (excluding no partner component)	0.161	0.051, 0.270	.004
Components of social isolation score (excluding living alone covariate)			
No partner	0.320	0.278, 0.362	<.001
Children (less than monthly contact)	0.037	−0.003, 0.077	.073
Other family (less than monthly contact)	0.015	−0.032, 0.063	.530
Friends (less than monthly contact)	0.044	0.004, 0.083	.029
No organizational membership	0.087	0.039, 0.136	<.001

*Notes:* ATT = average treatment effect on the treated; CI = confidence interval.

By removing the living alone covariate, housing with care residents appear at even higher risk of social isolation than would be expected if they were living in the community, with an ATT of 0.513 (95% CI 0.400, 0.626). The result for the “no partnership” component alone also becomes significant when modeled without living alone as a covariate, with an ATT of 0.320 (95% CI 0.278, 0.362). The magnitude of the effect for “no partnership” alone is even higher than the estimate for social isolation when living alone was included. The interpretation of the ATT for “no partnership” is that housing with care residents are more likely to have no partner than they would if they were living in the general community. This finding makes sense, as the loss of a partner can be a key driver of moves into such housing.

However, by excluding living alone from our matching covariates, we potentially lose a key characteristic that shapes the social experiences of later life. This is even more potent when considering the vast difference in prevalence of living alone in our sample compared with ELSA: the rates are figurative mirrors at 79% and 23%, respectively. Consequently, the changes in ATT when excluding living alone are likely to result from a worse matching of respondents rather than any adjustment for correlation between partnership status and living alone. The higher ATT when excluding living alone is therefore likely to be an overestimate of the effect of housing with care residence on social isolation compared to the previous result including it.

## Discussion and Implications

Our findings provide compelling evidence that people living in housing with care experience lower levels of loneliness than if they were living in the general community. There is also evidence that residents score slightly higher for social isolation than if they were living in the community, driven by less frequent contact with friends and social organizations. Taken together, these findings underscore the importance of looking at loneliness and social isolation as distinct (although interrelated) experiences rather than two versions of the same concept.

Our findings suggest that housing with care schemes have a positive impact on residents’ subjective experiences of social connections as reflected through loneliness. Given that loneliness has been associated with poorer health outcomes, which in turn are linked to increased dependence, these results strengthen the evidence that housing with care can play a positive role in an aging society. Not only can these schemes boost older people themselves through maintaining independence and autonomy, but such impacts will have knock-on benefits for health and care systems through reduced demand. This reinforces the case for investment in such properties and providing support for older people to move into similar housing.

In contrast to the results for loneliness, we found that social isolation may be slightly higher for housing with care residents, implying they are losing some degree of their social networks when they move in. Our analysis shows that this is driven mainly by less frequent contact with friends and lower levels of organizational membership. This may not be surprising, as relocation of any sort can disrupt interactions with friends and social groups based in the local community. Social bonds with family may be stronger and therefore less disrupted by relocation. It may also be that people who move into housing with care are more likely to have experienced declines in their friendship networks due to death, which is not captured in the covariates used in our modeling. Alternatively, respondents may have replaced contact with friends with contact with other residents but not considered other residents when answering about friends—akin to the concept of neighbors or acquaintances rather than friends—with some evidence this may be the case in residential settings for older people (Abbott et al., 2000). In addition, respondents could be active in the social groups available at their residence, which may not have been captured in responses around organizational membership. This suggests that measuring social isolation in housing with care settings may require context-specific measures to capture the full range of social interactions.

At the same time, we found no evidence that contacts with family were any different for housing with care residents than if they were residing in the community. This suggests that family members remain just as engaged in the lives of residents after their move. By extension, this also implies that family members are not expressing increased concern for their loved one after a move.

To our knowledge, this is the first analysis of its kind to collect survey responses on social connections across a sample of housing with care residents in a way comparable to other large-scale surveys. Here, we have facilitated a comparison with older people living in the general community. Using the PSM technique provides insight into the effect that residence in housing with care has independent of other factors, allowing a counterfactual assessment of the impact of such schemes on social connections in later life.

There are some limitations in our analysis. Without detailed information on the complete representative population of housing with care residents, we could not develop weights to account for differential nonresponse. It is reasonable to expect that residents with more severe chronic illness, high levels of isolation, or other barriers (such as visual impairment or dementia) could have been less likely to participate in our survey. While we made efforts to enhance inclusion, it is unclear if these were sufficient to capture responses from the most vulnerable. Our sample may be biased toward residents with higher levels of functional capacity, which may be further impacted by our response rate, restricting our ability to generalize from our survey to the overall population of people living in housing with care in England and Wales. That said, 85% of our sample report an activity-limiting condition, so we might speculate that our findings reflect a level of low capacity typical in housing with care settings.

This does not, however, negate our analytical findings in making a matched comparison between those living in housing with care and in the community. The use of PSM applies matching with respect to the covariates included, while reporting the ATT (as opposed to the average treatment effect or ATE) captures the counterfactual results of our sample members (housing with care residents) compared with what their outcomes would have been were they to differ only in terms of general community residence. In other words, the matching approach remains robust independent of representativeness in our resident sample. One potential limitation comes from the matching covariates used, as other factors may relate to whether people live in housing with care.

Both our survey and ELSA have low numbers of people from social minority backgrounds, which is another drawback. We know that people from such groups may face additional barriers to social inclusion. Our project explored the element of diversity in more detail through qualitative research, but the survey-based analysis here can provide no insights in this respect. In other words, we cannot say whether the effects of housing with care residence on loneliness and social isolation we have found would apply to people from social minority backgrounds. This remains a critical consideration for future investigation.

The contrast between the findings for loneliness and social isolation likely points to important lessons for stakeholders engaged in the later-life housing sector. Providers of housing with care can be proud that the subjective experiences of residents are improved with respect to loneliness. They could, however, explore ways to encourage residents to maintain links with their friends and social groups external to the scheme or facilitate similar connections within schemes. This will rely on active engagement with residents to hear about their experiences and identify factors that hinder social inclusion.

Our work also has implications for the UK government as part of its efforts to address loneliness. Our research contributes to a key objective of their 2018 loneliness strategy to improve the evidence base around loneliness. Our research highlights the overlap in housing, health, and aging policy and how action to expand housing with care could have benefits beyond just homes, linking to a second objective of the strategy to ensure loneliness is addressed across policymaking. The government should consider the kinds of incentives that are necessary to make proposals for new schemes more attractive to developers and providers and to encourage older people to move into such schemes.

Ultimately, housing with care schemes are homes for the people who live there. They should therefore be welcoming, inclusive spaces for all. Despite a slight reduction in some social contacts, our research has shown positive impacts on residents’ subjective social experiences from housing with care residence.

## Supplementary Material

igac061_suppl_Supplementary_Material
