# Supplementary material for: The Impact of Living in Housing With Care and Support on Loneliness and Social Isolation: Findings From a Resident-Based Survey
Source: Innov Aging. 2022 Sep 29;6(7):igac061. doi: 10.1093/geroni/igac061 (PMC9703102; doi:10.1093/geroni/igac061)

**Online Supplementary Material: Methodological details**

1. Details on covariates used for propensity score matching

2. Balance plots

3. Checks on loneliness results: Standardized differences and variance ratios

4. Checks on social isolation results: Standardized differences and variance ratios

**1. Details on covariates used for propensity score matching**

Our matching was built using the following measures:

- Gender: We use gender as reported by respondents. An open-ended item asked if the respondent identified as transgender or used other words to describe their gender identity; only one survey respondent noted this, albeit under a separate question about sexual orientation.
- Age group: Five-year age groups were used, bookended by the groups under 55 and 90+. Due to low numbers, the under 55 group was dropped from analysis.
- Ethnicity: Respondents could select their ethnic or cultural background from six options or through an open-ended option. Write-in responses were recoded accordingly. Due to numbers, this indicator was reduced to a binary variable of white or not white.
- Self-reported health (SRH) status: Respondents were asked to say if their health was currently excellent, very good, good, fair, or poor.
- Long-standing illness or disability: Respondents were asked if they had any long-standing illness, disability, or infirmity, with long-standing defined as “anything that has troubled [them] over a period of time or that is likely to affect [them] over a period of time.” Answers were restricted to yes or no.
- Living alone: Respondents indicated whether they currently lived alone.
- Socioeconomic status: We drew on responses related to the socioeconomic classification of occupational category, i.e. higher, intermediate, or manual, based on the NS-SEC system in the UK, asking respondents to think about their previous experiences with work.

With respect to assessing socioeconomic status, we avoided asking about income directly in our survey, given the potential for response error due to self-completion or a possible disincentivising effect. We did ask if they received any housing benefit or allowance as a proxy for financial situation; our project advisory board and partner providers indicated that residents would understand this term and be able to answer. We first used this measure in our analyses, matched to state benefit recipients in ELSA, but the NS-SEC measure proved more robust in sensitivity analyses.

**2. Balance plots**

**3. Checks on loneliness results: Standardized differences and variance ratios**


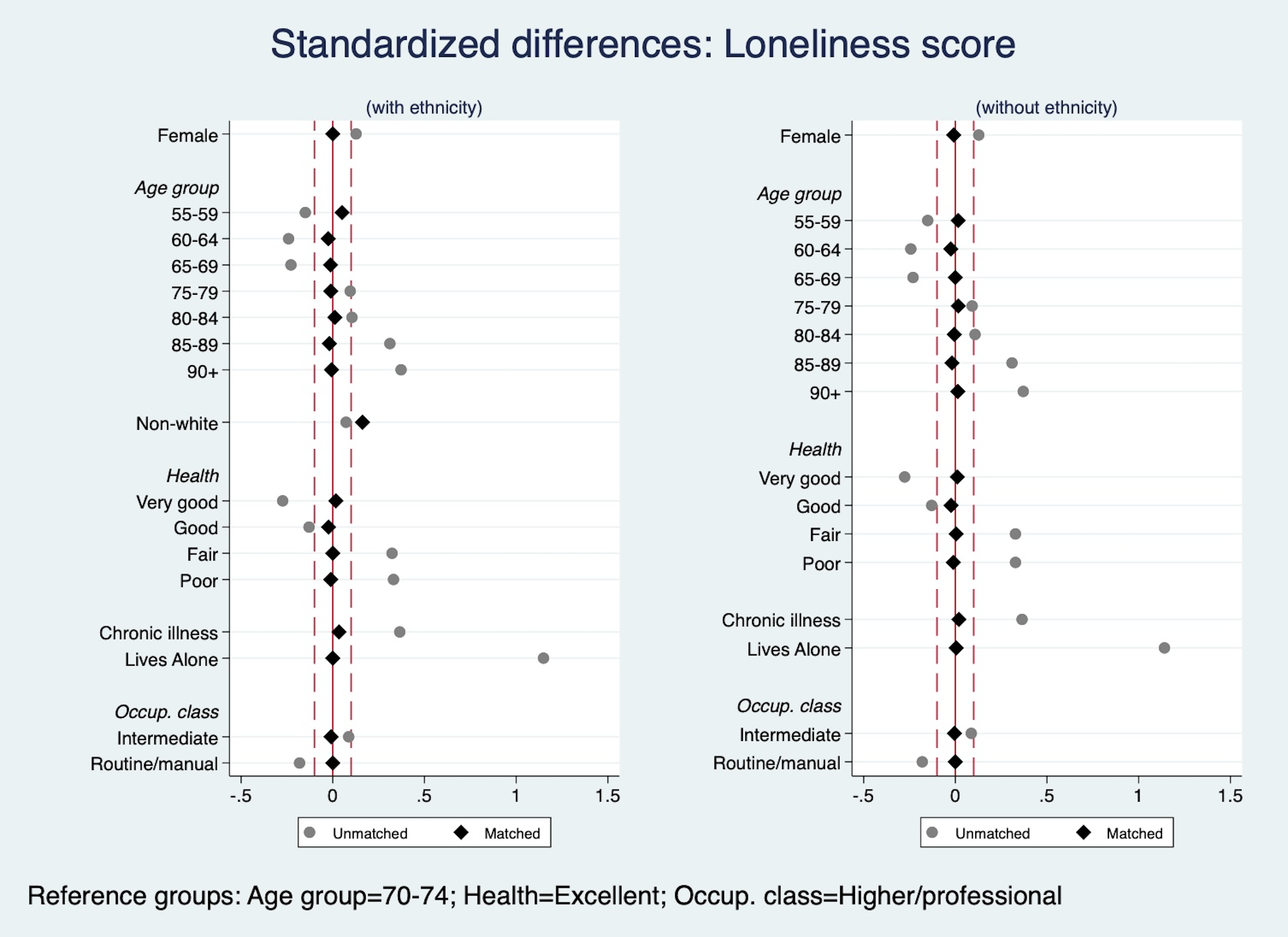


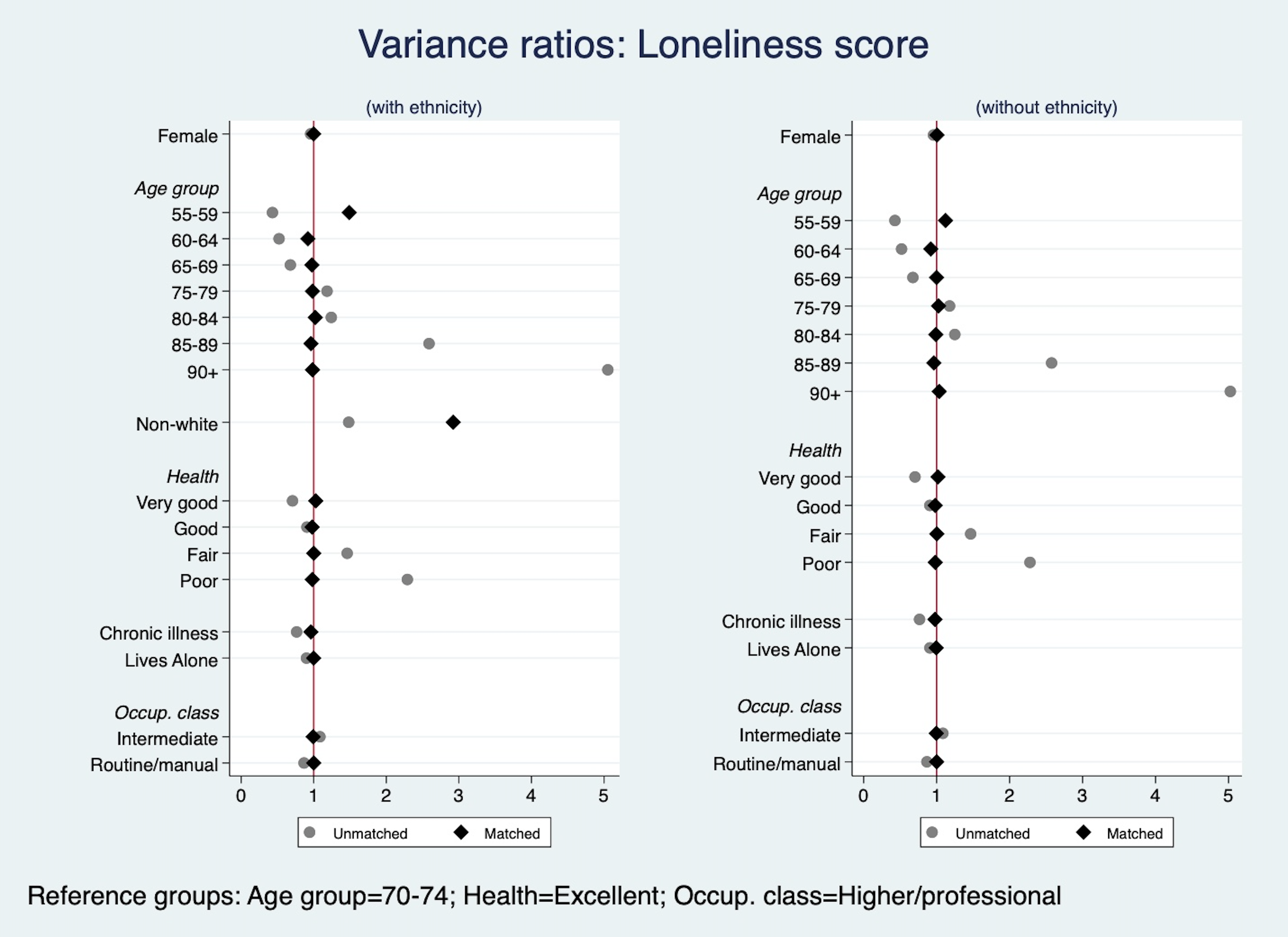


**4. Checks on social isolation results: Standardized differences and variance ratios**


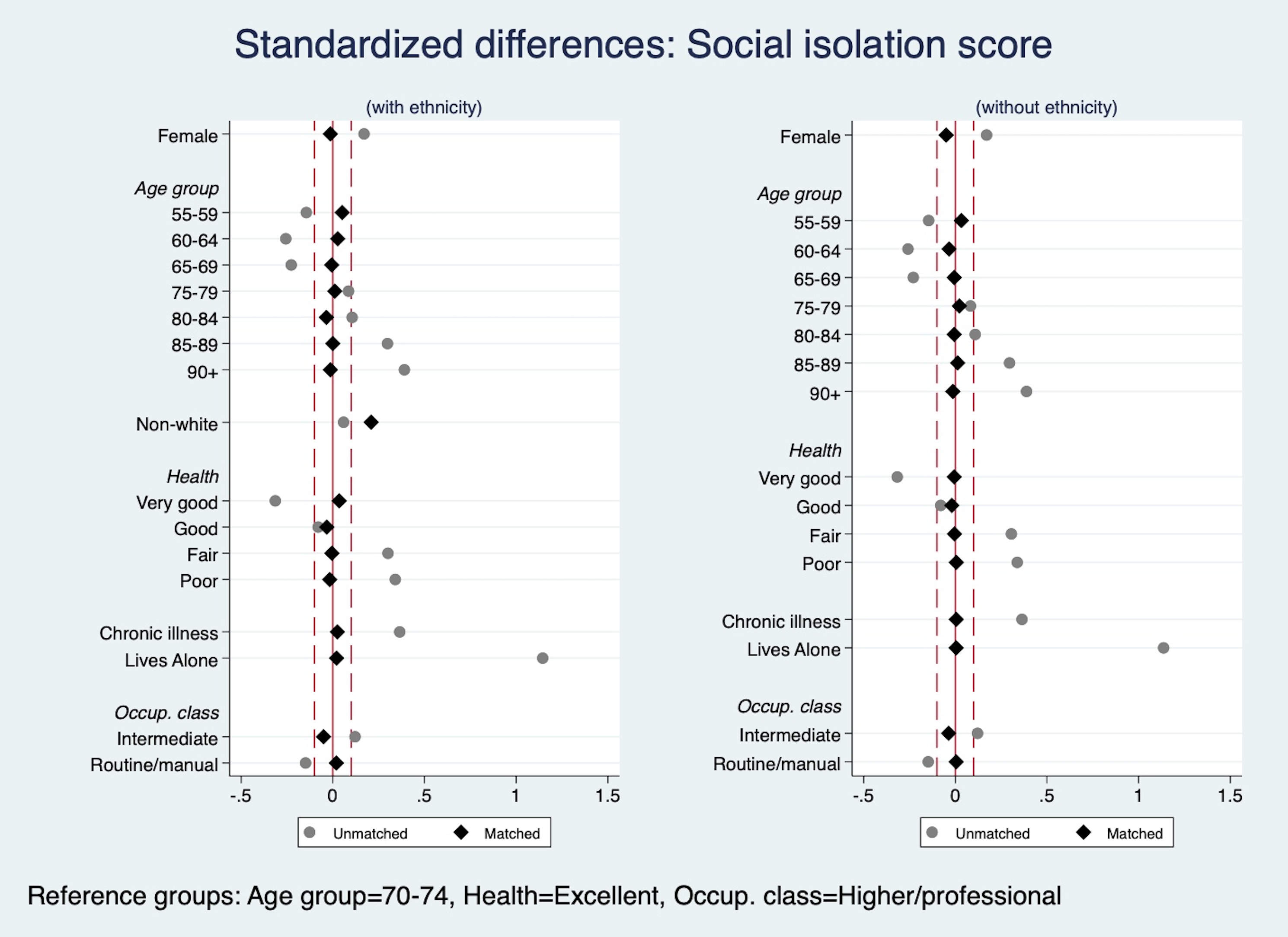


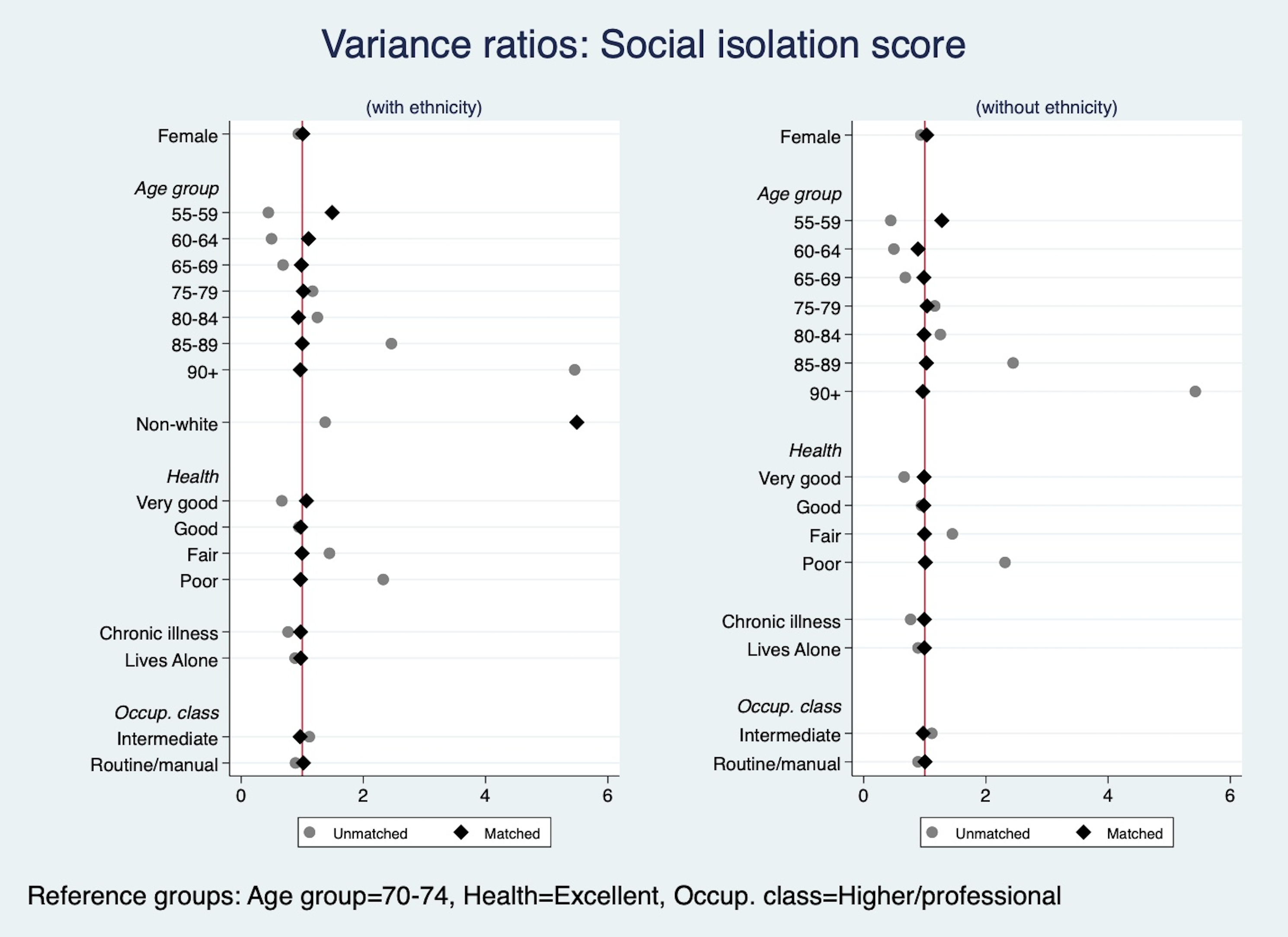

Supplement: igac061_suppl_Supplementary_Material [file igac061_suppl_supplementary_material.docx]
